# Hydatid disease of scapula and upper third of humerus treated by en bloc excision and fibular bone grafting

**DOI:** 10.4103/0019-5413.33691

**Published:** 2007

**Authors:** P Ranga Chari

**Affiliations:** Department of Orthopedics, Osmania Medical College, Hyderabad - 500 001, India

**Keywords:** En bloc excision, hydatid disease, scapula and humerus

## Abstract

35-year-old male patient presented with gradually increasing painful swelling of the right shoulder, which was incised and drained and wound persisted as a discharging sinus on the anterolateral aspect of the deltoid region with seropurulent discharge. A clinical diagnosis of tuberculosis of the shoulder was made. Plain skiagram of the right shoulder revealed multicystic lesion involving the entire scapula and upper third of the humerus with loss of joint space and pathological fracture at the junction of upper one-third and lower two-thirds of the humerus. A clinico-radiological diagnosis of hydatid disease was made. In view of the extensive involvement of the scapula with stiff shoulder and an active sinus, a two-stage surgical procedure was performed. Stage 1 consisted of en bloc excision of the scapula, upper half of the humerus and lateral end of the clavicle. Stage II surgery, consisting of fibular bone grafting. Tablet albendazole (400 mg, thrice daily) was given as systemic scolicidal agent. This case is reported in view of it's rarity and to highlight the management.

Hydatid disease of the scapula is uncommon. But hydatid disease of the scapula associated with the upper third of the humerus is even more rare. To the best of our knowledge there is no reported case of hydatid disease of the scapula associated with the upper third of the humerus. We treated this case by en bloc excision followed by fibular bone grafting for functional stability of the upper limb.

## CASE REPORT

A 35-year-old male school teacher presented with gradually increasing painful swelling of the right shoulder extending to the chest wall of two years duration. Suspecting it to be an abscess, an incision and drainage was performed by a rural practitioner six months back. Instead of pus there was a sudden gush of serous fluid through the incision. Since then the incision wound persisted as a discharging sinus. It was associated with low-grade fever. The patient gave history of discharge of a few small flakes of whitish tissue.

**Figure 1:**
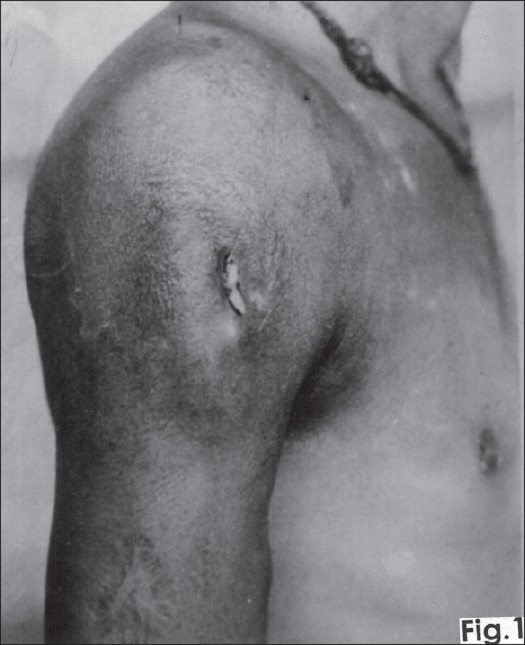
Clinical photograph showing right shoulder with diffuse swelling extending into pectoral region filling delto-pectoral groove and upper third of arm with a sinus in anterior-lateral aspect of deltoid region.

On clinical examination the swelling was prominent in the upper third of the right arm extending on to the pectoral and scapular region of the chest with fullness in the delto-pectoral groove. There was a sinus on the anterolateral aspect of the deltoid region with seropurulent discharge [Figure 1]. The skin around the sinus was darkly pigmented and indurated. The swelling was warm, tender and had variable consistency. All movements of the shoulder joint were severely restricted and painful. A clinical diagnosis of tuberculosis of the shoulder was made.

**Figure 2:**
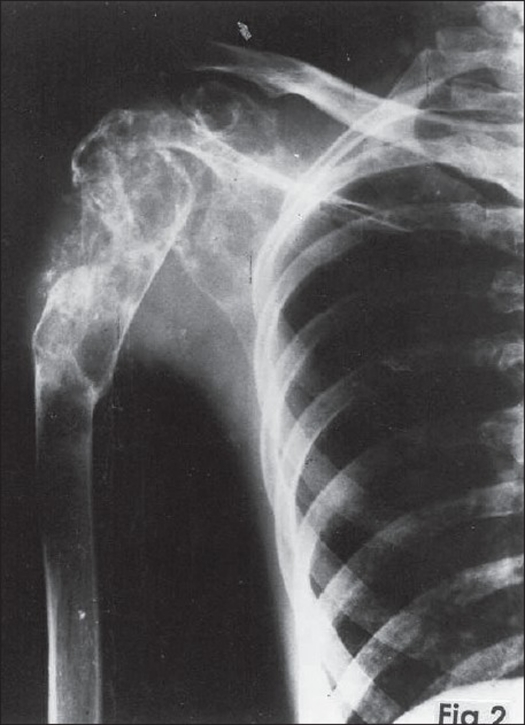
Plain skiagram of right shoulder showing expansile multicystic lesion involving the entire scapula and upper third of humerus. Note malunited pathological fracture of humerus.

Plain skiagram of the right shoulder revealed a multicystic lesion involving the entire scapula and upper third of the humerus with loss of joint space and pathological fracture at the junction of upper one-third and lower two-thirds of the humerus [Figure 2]. A clinico-radiological diagnosis of hydatid disease was made. All hematological investigations were normal. Repeatedly Casoni's test for hydatid disease was negative.

After controlling secondary infection with suitable antibiotics, a gentle diagnostic scooping through the sinus was done. A tiny pearl-like whitish cyst was found in the scrapings. Histopathology confirmed the diagnosis of hydatid disease.

**Figure 3:**
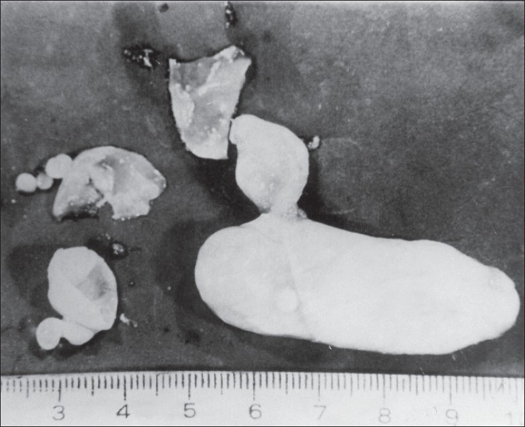
Photograph showing hydatid cysts dissected out of the specimen.

In view of the extensive involvement of the scapula with stiff shoulder and an active sinus, fore quarter amputation was advised. However, the patient refused an amputation and requested for a limb salvage procedure. A two-stage surgical procedure was planned due to the presence of secondary infection. Stage 1 consisted of en bloc excision of the scapula, upper half of the humerus and lateral end of the clavicle. On dissection the specimen revealed hydatid cysts [Figure 3].

The limb was splinted to the chest with well padded dressings. The patient was given albendazole tablets (400 mg, thrice daily) as systemic scolicidal agent. After four weeks, the limb was suspended in an arm sling.

**Figure 4:**
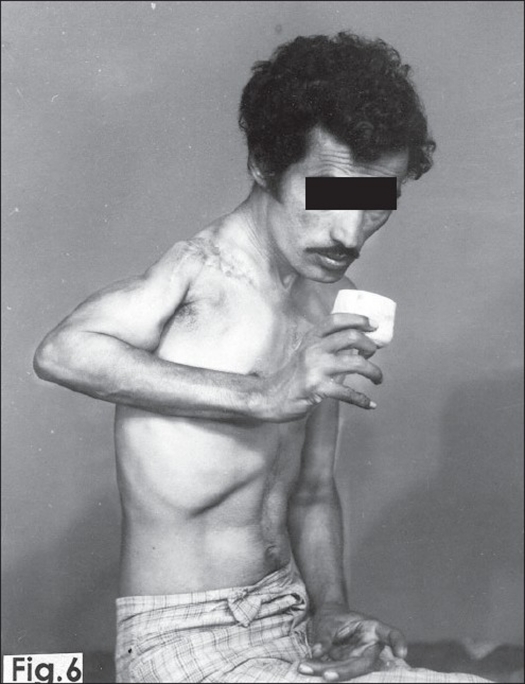
Follow up clinical photograph shows 30°-40° of active painless movements at shoulder region with reasonably good stability

**Figure 5:**
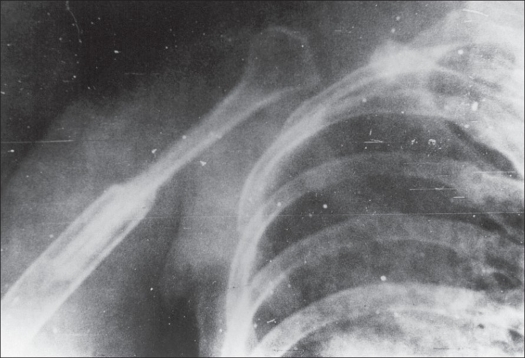
Plain Skiagram of right shoulder shows fibular graft in the remaining distal humerus showing consolidation of the graft

Five weeks later stage II surgery, consisting of fibular bone grafting, was performed through a vertical delto-pectoral incision. The deltoid and pectoralis major muscles were separated. The osteotomized end of the upper three-fourth of the fibula, taken from the right leg, was pegged into the medullary canal of the humerus and its head was fixed within the deltoid muscle nearer to its origin. The upper end of the fibula, with deltoid muscle, was fixed to the first rib in the mid-axillary line with non-absorbable nylon sutures. The wound was closed after thorough hemostasis and the upper limb was immobilized in POP shoulder spica, keeping arm and forearm as in shoulder fusion, for six weeks. After removing the shoulder spica, the limb was suspended in arm sling and gentle pendulum movements were started. After two months of physiotherapy patient regained about 30°-40° of active painless movements at shoulder region with reasonably good stability [Figure 4]. After four months of mobilization the patient was able to lift 3-4 kg weight. He could eat and write with his right hand. There was no recurrence of disease after one year of surgery with consolidation of fibula with host bone [Figure 5]. The patient was subsequently lost to follow-up.

## DISCUSSION

To the best of our knowledge there are no recorded reports of hydatid disease involving the entire scapula and upper one-third of the humerus. Out of 637 cases of hydatid disease of bones reported in 1948, only 10 cases had scapular involvement.^1^ Further 40 cases of hydatid disease of bone were reported in 1964, out of which only one patient had lesion in the vertebral border of the scapula.^2^

Total scapulectomy was considered as a limb salvage procedure to forequarter amputation for certain malignant conditions of the scapula. Since then there have been many reports discussing the operative procedure and their functional out com of results.^3^–^8^ Rush reported hydatid disease of the scapula as another indication for total scapulectomy. He reported a case of hydatid disease of the scapula treated successfully by total excision of the scapula.^1^
